# Surgical Aspects of Treatment of the Lung Cancer Found in Low-Dose CT-Based Screenings †

**DOI:** 10.3390/jcm15030947

**Published:** 2026-01-24

**Authors:** Małgorzata E. Wojtyś, Janusz Wójcik, Arkadiusz Waloryszak, Norbert Wójcik, Piotr Lisowski, Tomasz Grodzki

**Affiliations:** 1 Department of Thoracic Surgery and Transplantation, Pomeranian Medical University in Szczecin, Alfreda Sokołowskiego 11, 70-891 Szczecin, Poland; 2 Clinic of General, Minimally Invasive and Gastroenterological Surgery, Pomeranian Medical University in Szczecin, 71-252 Szczecin, Poland; 3 Student Scientific Club of Thoracic Surgery and Transplantation, Pomeranian Medical University in Szczecin, 70-204 Szczecin, Poland

**Keywords:** lung cancer, low-dose computed tomography (LDCT), screening, pilot study, thoracic surgery department

## Abstract

**Background:** Lung cancer is the leading cause of cancer-related death worldwide. Screening with low-dose computed tomography (LDCT) enables early detection of low-stage non-small cell lung cancer (NSCLC), increasing the chances of curative surgery. The aim of the present study was to analyze selected surgical aspects of treatment among patients diagnosed with NSCLC through LDCT-based screening in Szczecin, the first program of this kind in Poland. **Methods:** A group of 52 patients who were screened and operated on was compared with patients diagnosed and operated on outside the screening program during the same time period and a group of patients diagnosed and operated on prior to the screening program being implemented. **Results:** The screened population demonstrated a significantly higher frequency of stage IA cancer diagnosis, smaller tumor volume, more lobectomies, and fewer pneumonectomies compared with the other two groups. In addition, the waiting time for surgery was shorter, the duration of the procedure longer, and the length of hospitalization was reduced among the screened patients. No significant differences were observed in postoperative mortality or perioperative complications. Adenocarcinoma occurred significantly more often in the screened population than in the other groups, and tumors were more frequently classified as grade G2. A significant correlation was found between the need for blood transfusion and the occurrence of perioperative complications. **Conclusions:** The implementation of an LDCT-based screening program for lung cancer has a significant impact on the workload and case profile of thoracic surgery departments. Several aspects of surgical treatment differ significantly between patients diagnosed through screening and patients diagnosed outside of the program.

## 1. Introduction

Lung cancer remains the leading cause of cancer-related mortality worldwide, regardless of gender or ethnicity. In terms of morbidity, lung cancer ranks second in men and in women in the United States [1]. In the European Union, lung cancer causes approximately 270,000 deaths per year [2,3]. Poland is among the countries with the highest morbidity and mortality from lung cancer [4,5,6,7]. Lung cancer has a very poor prognosis. Primary prevention (i.e., reducing or ceasing smoking and avoiding passive smoking) is thought to be the only effective approach. Lung cancer is often clinically silent when confined to the lung parenchyma, and it causes non-specific symptoms when growing in the bronchi. A reported 30–57% of patients have distant metastases at the time of diagnosis [2,8].

Computed tomography (CT) provides sufficient resolution to detect small lung nodules. Low-dose non-contrast CT has gained interest as a screening tool for early lung cancer detection while minimizing radiation exposure. Early diagnosis increases resectability and eligibility for curative surgery, which remains the most effective treatment for non-small cell lung cancer but is limited to early-stage disease. Lung cancer has low resection rates (16–20% in Poland [9,10] and ~30% in countries with more advanced health care systems [7]), largely due to late diagnosis [11]. It also has one of the lowest 5-year survival rates among malignancies (14–18%) [4,8]. Five-year survival reaches approximately 67% in stage I disease but drops to 23% in stage III [12]. Only 15–25% of cases are diagnosed at a stage suitable for radical surgery [13], while over 60% are detected at advanced stages [14,15]. Early detection can increase 5-year survival to over 50% [2].

In 2011, the National Lung Screening Trial (NLST) reported for the first time a significant decrease (20%) in lung cancer mortality among a group of patients undergoing screening by LDCT compared to a group of patients diagnosed using chest X-ray [16,17]. Among patients operated on for stage I lung cancer, 93% remained relapse-free for 5 years. In 2016, the NELSON study (Dutch-Belgian Randomized Lung Cancer Screening Trial) conducted in the Netherlands and Belgium showed a significant decrease in lung cancer mortality (26% in men and 39–61% in women) among patients screened by LDCT compared with patients who were not screened [3,18,19,20,21,22,23].

Although the research data may seem promising in terms of reducing mortality, questions remain regarding the implementation of universal screening for high-risk individuals, primarily regarding cost-effectiveness and overtreatment.

Lung cancer screening programs worldwide vary in their inclusion criteria, including age, tobacco exposure, environmental or occupational exposure, chronic respiratory disease, and family history of lung cancer. Smoking remains the dominant eligibility criterion in most programs [24], as the most significant risk factor. Data shows that 1 in 10 smokers develops lung cancer an average of 30 to 40 years after they start smoking [25]. Some authors have stated that screening programs for early lung cancer detection prove to be more effective when combined with a program supporting smoking cessation [26,27]. The United States Preventive Services Task Force recommends annual screening for adults aged 50 to 80 years with a history of at least 20 pack-years of cigarette smoking. Former smokers must have quit smoking within the past 15 years to be eligible [28].

A study from Greece suggested that implementing an LDCT screening strategy covering 100% of high-risk adults aged 50–80 years would avoid additional deaths and increase the lung cancer life years (LCLY) over 5 years [3]. Similar conclusions were drawn in studies in Japan [8]. The Lithuanian screening program examined individuals aged 50 to 70 years. This program was unique in that it included individuals regardless of smoking status. The results of this study also indicated that diseases would be detected before they become clinically apparent in many individuals through a single, non-invasive, periodic examination. Furthermore, disease detection strongly motivates patients to change harmful habits and take preventive measures [2]. Another study [29] involved elderly participants (aged ≥ 70 years), a population that often fails to meet the stringent inclusion criteria of clinical trials. The findings indicated that early detection and timely initiation of appropriate therapy are achievable in this group, with nearly half of the patients undergoing radical surgery at an early stage of the disease.

This study analyzed the surgical aspects of non-small cell lung cancer detected through a low-dose CT–based screening program in Szczecin, the first program of its kind in Poland. The aim was to evaluate the impact of introducing LDCT screening on the surgical management of non-small cell lung cancer and on the functioning of a thoracic surgery department. The analysis included patient and lesion characteristics identified through the program, as well as perioperative care and organizational aspects related to hospitalization.

## 2. Materials and Methods

### 2.1. Patients

The included patients underwent surgical treatment for lung cancer in the Thoracic Surgery and Transplantation Clinic of the Pomeranian Medical University in Szczecin. The protocol was based on the I-ELCAP and NELSON programs. This retrospective study was based on the available medical records and data from the screening program’s database.

The patients were divided into three groups: Group 1a included 52 surgically treated lung cancer patients participating in the LDCT-based early lung cancer screening program in April 2008 to December 2009; Group 1b included 87 patients surgically treated for lung cancer during the same period as Group 1a but were not included in the program; and Group 2 was the control group of 103 lung cancer patients treated surgically in the period prior to the program’s implementation (July 2006–March 2008).

The surgeries were performed in 2006–2009 via thoracotomy. The extent of the resection ranged from wedge resection through lobectomy and bilobectomy to pneumonectomy. The extent of the operation depended mostly on the cancer stage.

### 2.2. Screening

Lung cancer early detection screening based on low-dose CT was first conducted in Poland in Szczecin in April 2008. The patients qualified for screening if professionally active, aged 55 to 65 years, and smoking currently or had a history of at least 20 pack-years of smoking, regardless of gender. From the beginning of April 2008 to the end of December 2010, a total of 15,017 tests were performed within the program. Lung cancer was diagnosed in 107 cases, giving an occurrence rate of 7.1 in 1000 test cases. In the group of operated patients participating in the program (i.e., Group 1a), 68.8% of all lung cancers were in stage I.

### 2.3. Statistical Analysis

For categorical variables, a two-count comparison test with correction and a chi-squared independence test with the Yates correction were used. For discrete and continuous variables whose distribution was not normal, the non-parametric Mann–Whitney U test was used. The parametric Student’s *t*-test was used for continuous variables with a normal distribution. The significance level was assumed to be 0.05. Statistical analyses were carried out using the Statistica PL 2010 program.

## 3. Results

### 3.1. Comparison of the Study Groups in Sex and Age

The patients were divided into three groups: Group 1a included 52 surgically treated lung cancer patients participating in the diagnosis program; Group 1b included 87 patients surgically treated for lung cancer outside the program but during the same period; and Group 2 was the control group of 103 lung cancer patients treated surgically in the period prior to the program’s implementation. The sex and age distributions of the groups are given in Table 1.

### 3.2. Comparison of the Study Groups Regarding Smoking

Smoking was considered one of the most important risk factors for lung cancer. All patients in Group 1a were cigarette smokers, as smoking was a factor for inclusion in the screening study. In Group 1b, 74 individuals (85.1%) were smokers, and 91 individuals (88.3%) in Group 2 were smokers. No significant relationship was found among groups.

### 3.3. Distribution of Cancer Locations in Each Group

The location of primary lung cancer in each group is illustrated in the table below (Table 2).

### 3.4. Histopathological Types

**Figure 1:**
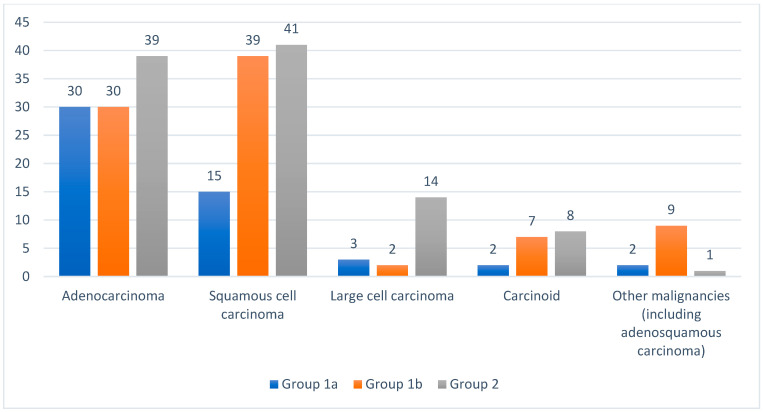
Types of primary lung cancer in the three study groups based on the histopathological diagnosis.

Figure 1 illustrates the types of primary lung cancer based on the histological diagnosis. Adenocarcinoma had a significantly higher occurrence rate in Group 1a than in Group 1b (*p* = 0.012) and Group 2 (*p* = 0.0297). No significant difference in the occurrence rate of adenocarcinoma was found between Groups 1b and 2 (*p* = 0.740). Comparing the occurrence rates of squamous cell carcinoma, large-cell carcinoma, and carcinoid, no significant relationship was found between Groups 1a and 1b or between Groups 1a and 2. However, when comparing groups 1b and 2, a statistically significant difference was found in the incidence of large cell carcinoma (*p* = 0.01).

### 3.5. Grading of the Primary Lung Cancer

The distribution of lung cancer grades in each group is shown in Table 3. A significant difference in the occurrence rates of grades G2 and G3 was found in Group 1a (*p* < 0.001), with G2 occurring more frequently. However, the differences in the occurrence rates of G1 and G2 or G1 and G3 were not significant. No significant differences in grades were found in Groups 1b and 2.

Comparing occurrence rates between Groups 1a and 1b, the differences were not significant. We also found no significant differences when comparing Groups 1b and 2. In contrast, we found a significant difference in grade G2 occurrence between Groups 1a and 2, as it occurred significantly more frequently in Group 1a (*p* = 0.02). However, the difference in the occurrence of G1 and G3 was not significant between these two groups.

### 3.6. Lung Cancer Staging

The TNM VII classification for each group is given in Table 4. Comparing Group 1a to Groups 1b and 2, only the T1a feature, carcinoma of the lowest stage, was significantly different (*p* = 0.002 and *p* = 0.001, respectively). This difference was not found between Groups 1b and 2. Table 5, Table 6 and Table 7 present the comparison among groups. Regarding clinical stages (IA-IV based on the TNM VII classification—Table 8), IA occurred significantly more frequently in Group 1a than in Group 1b (*p* = 0.03) and Group 2 (*p* = 0.025), whereas IIIA occurred significantly more frequently in Group 1b than in Group 2 (*p* = 0.03).

### 3.7. Other Studied Factors

Tumor volume was measured in cubic centimeters (cm^3^) and was significantly smaller in Group 1a than in Group 2 (*p* = 0.01) and Group 1b (*p* = 0.004), as well as both Groups 1b and 2 combined (*p* = 0.003)—Table 9. No significant difference was found in tumor volume between Groups 1b and 2 (*p* = 0.53).

The waiting time for surgery was significantly different between Groups 1a and 2 (*p* = 0.0134), but not between Groups 1a and 1b (*p* = 0.39) or Groups 1b and 2 (*p* = 0.08).

A significant difference in the mean procedure duration was found between Groups 1a and 1b (*p* = 0.02) and Groups 1a and 2 (*p* = 0.007). The average procedure duration was longer in Group 1a than in the other groups, but we did not find a significant difference in the average durations of procedures in Groups 1b and 2. The duration of surgery in patients with complications (*n* = 48 patients) and without complications did not differ significantly (*p* = 0.79).

Groups 1a and 1b had significantly shorter duration of hospitalization than Group 2, but no significant difference was found between Groups 1a and 1b. Therefore, the duration of hospitalization was significantly shorter during the screening program than in the period before the program was implemented. However, during the period of the early lung cancer detection program, there was no significant difference between patients who did and did not participate in the program.

### 3.8. Invasive Procedure-Based Diagnosis

The number of patients diagnosed via an invasive procedure is provided in Table 10.

No significant difference was found among the groups. However, a diagnosis was not made based on invasive procedures approximately 1.6-times more often in Group 1a.

### 3.9. Types and Numbers of Performed Procedures

**Figure 2:**
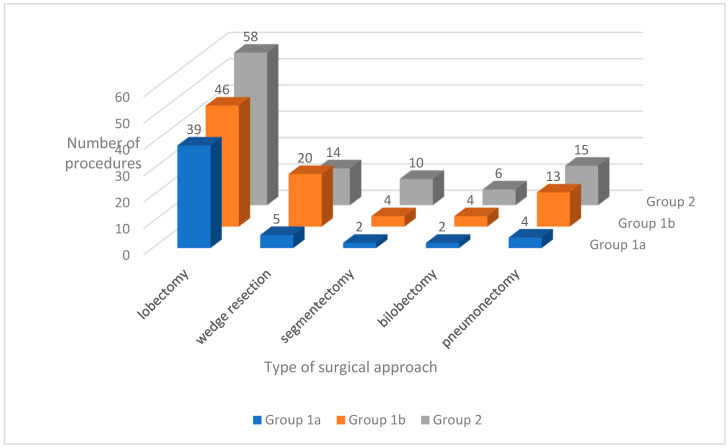
Types of surgical approaches in each group.

The distribution of surgical approaches used in each group is shown in Figure 2. A comparison of the procedures performed is provided in Table 11.

Of all the procedures, only lobectomy with mediastinal lymphadenectomy was significantly more frequent in Group 1a than in Groups 1b and 2.

Significantly more surgeries due to primary lung cancer were performed (*p* < 0.05) during the period of the screening program compared with the period before the program.

### 3.10. Perioperative and Postoperative Complications

We found no significant difference in the occurrence of such perioperative complications between Groups 1a and 1b (*p* = 0.25), Groups 1a and 2 (*p* = 0.12), or Groups 1b and 2 (*p* = 0.80). We also found no significant differences in the frequency of perioperative deaths between Groups 1a and 2 (*p* = 0.53), Groups 1a and 1b (*p* = 0.71), or Groups 1b and 2 (*p* = 0.85).

In Group 1a, a late postoperative complication occurred in 1 (1.9%) patient in the form of chronic emphysematous space. In Group 1b, no late postoperative complication occurred. In Group 2, a late postoperative complication occurred in 4 (3.9%) patients: wound dehiscence with subcutaneous hematoma that required reoperation, acute respiratory failure and circulatory failure, wound dehiscence and empyema of the right pleural cavity, and empyema requiring the Weder method.

No significant relationships were found among the groups for the frequency and grading (Group 1a, *p* ≥ 0.32; Group 1b, *p* ≥ 0.36; Group 2, *p* ≥ 0.47) and co-morbidities (Group 1a, *p* = 0.75; Group 1b, *p* = 0.23; Group 2, *p* = 0.39). The duration of the procedure in the case of complications and without perioperative complications did not differ statistically significantly (*p* = 0.79).

However, a significant correlation was found between the need for blood transfusion and the occurrence of perioperative complications (all studied groups, *p* = 0.0002; Group 1b, *p* = 0.01; Group 2, *p* = 0.04). Of those patients who received transfusions, 39.3% experienced perioperative complications. Among those who did not require transfusions, 14.7% experienced perioperative complications. However, for Group 1a, this correlation was not significant (*p* = 0.6).

## 4. Discussion

The results of our study are consistent with large, randomized trials; our work provides additional important information. The literature on lung cancer screening seldom discusses the surgical and organizational issues encountered during the treatment of patients diagnosed over the course of these screenings [4,9].

Among the patients screened at our clinic, adenocarcinoma occurred significantly more frequently than in the other study groups. A CT of the thorax visualizes peripheral structures of the lungs, where adenocarcinoma is most frequently located, better than the central structures [30,31]. In central lung regions near the hilum, tumors may mimic vascular structures or lymphadenopathy. Squamous cell carcinomas typically occur centrally, whereas adenocarcinomas are more often peripheral. Thus, CT imaging is more effective in diagnosing peripheral lung lesions, and screening with LDCT tends to detect predominantly peripheral tumors, as observed in the LUSI trial and the American NLST [32,33].

Tumor size/volume is conceptually related to prognosis and is reflected in the T stage of the TNM system. The tumor volume is also an independent and important prognostic factor for 5-year survival. It provides a continuous and more detailed measure than categorical T staging, which may offer additional prognostic value. Patients diagnosed during the screening had significantly smaller neoplasms. For adenocarcinoma of the lung with a diameter of 10 mm measured on CT, the 5-year survival is estimated to be 97.9%, whereas estimates are 68.1% for diameters of 10–20 mm, 53.7% for diameters of 20–30 mm, and only 15.7% for diameters > 30 mm [34]. Tumor volume measurement in LDCT improves individual clinical decision-making. Automated volumetric nodule classification enhances assessment of growth dynamics (stable vs. growing), reduces radiologists’ workload, and has been shown to decrease false-negative classifications when AI is used as a standalone CT reader [35].

The significant differences in TNM VII clinical stage classification were similarly reported by other studies [34,36,37]. Invasive procedures (bronchofiberoscopy, Endobronchial Ultrasonography (EBUS), Endoscopic Ultrasonography (EUS), biopsy, and mediastinoscopy) were performed to confirm staging or diagnose indeterminate CT findings [38,39]; 42–77% of the NELSON program patients underwent invasive procedures to determine the lung cancer grade [40]. The current aim is to limit the number of invasive procedures in the patient population undergoing screenings for early lung cancer diagnosis [41,42,43].

The difference in the waiting time for surgery (from admission for surgery to the day of surgery) was significantly shorter in the screened population than in the period before screening, but we did not find a difference between the screened population and the patients who did not undergo screening during the same time period. In two studies that did not screen patients, one determined the waiting time to be 25 days, and the other reported 5 weeks [44,45].

We found a significant increase in the frequency of surgeries due to primary lung cancer (for all extents of pulmonary parenchyma resection) during the LDCT-based screening program compared with the period prior to the program’s introduction. The extent of the operation depended mostly on the cancer stage [46]. During the screening program, it seemed that the information from the LDCT pilot study would help identify a larger group of patients with primary lung cancer treated with traditional open lobectomy with mediastinal lymphadenectomy, which was the commonly used resection method those days. The experience accumulated over the following years enabled not only the implementation of LDCT screening but also the routine adoption of videothoracoscopic techniques, the broad application of anatomical sublobar resections, the use of combined CT and PET-CT imaging, the evaluation of EGFR mutations and PD-L1 expression, and the advancement of liquid biopsy technology. During the program of our study, the number of lobectomies with mediastinal lymphadenectomy was significantly greater in the screened population, whereas the number of pneumonectomies was roughly 50% lower in this group than in the other groups. In the Danish screening study, lobectomy was the most common procedure [47]. In the treatment of low-stage non-small cell lung cancer, lobectomy with mediastinal lymphadenectomy fulfills the definition of radical oncological resection. Performing resections more limited than lobectomy remains controversial [48]. A different opinion was presented by Hennon and Yendamuri, who stated that segmentectomy is sufficient for the radical resection of peripheral T1 masses with a diameter < 2 cm [49].

Surgeries were significantly longer in the screened population than in the other groups. In this group, 75% of the surgeries were lobectomies and, in some cases, an intraoperative examination was performed. Dominioni et al. reported an average operation time of 180 min in lung cancer patients, whereas the Danish screening study stated an average of 135 min (VATS) [47,50]. We did not find a correlation between the duration of the operation and the occurrence of perioperative complications, which contradicts the positive correlation reported by Shiono et al. [51].

Blood transfusions are considered an independent risk factor for perioperative complications and mortality. We confirmed a correlation between the number of transfused units and the occurrence of perioperative complications. Little et al. [52] reported that 13.7% of patients received blood transfusions and demonstrated a correlation between blood transfusion and an increase in perioperative complications and mortality. Thomas et al. showed that blood transfusion affected the mortality rate among pneumonectomy patients. They determined the factors causing the increased demand for blood transfusions, which affected 16–25% of patients [53]. Blood transfusions increase the frequency of infectious and respiratory complications due to transfusion-related acute lung injury [54].

The hospitalization time was significantly shorter during the period of screening than during the time period prior. Gargarine et al. reported an average duration of hospitalization of 7.3 days for patients undergoing lobectomy due to neoplasm and non-neoplasm-related reasons [55]. Alarcon et al. hospitalized their patients for 3.4 to 9.8 days on average and proposed a “Fast-Track Surgery” protocol to shorten it [48]. Shiono et al.’s patients were hospitalized for 13 days on average [51].

The screened population did not have any perioperative deaths, but the perioperative mortality reached 2.3% in the non-screened population operated on during the same time period, and was 2.9% prior to the screening period. The perioperative mortality (within 30 days from operation) rate among Little et al.’s patients was 5.2% after lobectomy, 8.5% after pneumonectomy, and 4.9% after peripheral resection [52]. Gagarine et al. reported a mortality rate of 2.2% for their lobectomy patients [55].

The screened population had the fewest number of complications. In addition, we identified a significant relationship between perioperative complications and blood product transfusions in the non-screened population and the time period before screening.

Beyond standard screening outcomes such as early detection, grading, and staging, this study focused on surgical aspects, including length of hospitalization and postoperative complications. Evaluating the screening program from a thoracic surgery perspective allowed assessment of its impact on departmental workflow and resource demands, with implications for clinical and economic planning. Previous studies have largely emphasized screening limitations, including high false-positive rates and indeterminate nodules, leading to additional diagnostic procedures, increased radiation exposure, misdiagnosis, which cause an overtreatment, exposing patients to unnecessary surgical procedures and patient anxiety. It is important to emphasize that, currently, routine preoperative evaluation of lung cancer should include additional computed tomography (CT). The purpose of this examination is to identify small metastases, lymph node involvement, and assess for involvement of other tissues and organs. Additionally, the lungs can be assessed to detect the presence or absence of comorbidities (emphysema, fibrosis, interstitial lung disease) and to examine anatomical variations in the bronchial vascular system [56]. Increasing screening specificity through automated nodule volume assessment and biomarkers can improve nodule identification [35]. Recent advances in biomarker research suitable for mass screening include proteins, miRNAs, circulating tumor cells, and tumor DNA [12]. As LDCT screening enables detection of early-stage disease, improved lung cancer risk models are increasingly valuable for patient selection [57,58]. Emerging approaches combined with LDCT aim to detect early malignancy, stratify risk, distinguish benign from malignant nodules, and predict treatment response, using blood-based biomarkers (cfDNA, methylation patterns, CYFRA 21-1, CRP), non-blood biomarkers (exhaled air compounds, sputum cells), and miRNAs to reduce the need for invasive testing [12,21,22,59].

Intensive work is currently underway on systems that assist in the interpretation of CT scans. Technological advances could take LDCT screening to a new level by automating the identification of nodules and reducing the number of false positives. Specialists are developing technological solutions, such as deep learning-based image reconstruction algorithms and artificial intelligence tools, to improve the quality of radiological images and help physicians cope with the increasing workload resulting from more CT scans by screening programs. Radiomics is based on the extraction of a large number of medical image features that support the identification of cancer-specific features using data characterization algorithms, and the use of numerous computer-aided diagnostic systems has led to the development of methods for faster and more accurate detection of lung nodules [35]. Improved methods for evaluating CT images have also been developed to minimize the risk of misinterpreting small changes in the lungs. In nodule detection, methods based on convolutional neural networks (CNN) are characterized by higher detection sensitivity and can be continuously improved and optimized based on LDCT scans [60,61,62]. The use of artificial intelligence for lung nodule detection has proven to be an effective and valuable tool in lung cancer screening, greatly supporting the diagnostic process, but current data indicate that a high rate of true negative diagnoses may still be a problem [63].

A limitation of this study is its pilot character and the restricted study population, as the screening was conducted in 2008 and included only residents of Szczecin. Strict inclusion criteria further limited the scope of analysis and precluded broader conclusions, such as those concerning age at first diagnosis. At the time of the study, limited access to diagnostic tools and procedures influenced both screening and surgical practices. Moreover, the analyzed medical records did not allow assessment of the financial aspects of implementing the screening program. These limitations indicate the need for further development of LDCT-based screening programs, involving larger and more diverse populations, and for analysis of outcomes using current diagnostic and therapeutic standards in lung cancer.

## 5. Conclusions

The implementation of an LDCT-based lung cancer screening program had a substantial impact on the surgical management of non-small cell lung cancer and on the functioning of a thoracic surgery department. Screening was associated with a higher number of surgical resections performed due to primary lung cancer than in the period prior to the screening being introduced. There was more frequent detection of early-stage and small-volume tumors with fewer pneumonectomies and more lobectomies with mediastinal lymphadenectomy. The introduction of screening was associated with shorter waiting times for surgical treatment without an increase in perioperative complications or mortality, despite longer operative times. The analysis of blood transfusions in our patients confirmed that there is a significant correlation between the transfusion and the perioperative complications.

These findings suggest that LDCT screening not only enables earlier diagnosis but also favorably influences surgical strategy and departmental organization in thoracic surgery. Such a program may lead to more frequent diagnoses of lung cancer at earlier stages, identification of smaller tumors, shorter waiting times for surgical intervention, and hospitalization.

## Figures and Tables

**Table 1:** Age and sex distributions of the three study groups.

	Group 1a	Group 1b	Group 2
Male, *n* (%)	23 (44.2%)	54 (62.1%)	59 (57.3%)
Female, *n* (%)	29 (55.8%)	33 (37.9%)	44 (42.7%)
Average age (range), years	60.2 (55–65)	65.2 (23–84)	62.7 (18–84)
SD for age	3.4	11	10.2

SD—standard deviation.

**Table 2:** Location of the cancer in each group.

Location		Number (%) Group 1a	Number (%) Group 1b	Number (%) Group 2
The right lung	Upper lobe	20 (58.8)	26 (50)	40 (69.0)
	Lower lobe	11 (32.4)	22 (42.3)	14 (24.1)
	Middle lobe	5 (14.7)	2 (3.8)	2 (3.4)
	Lung hilum	2 (5.9)	5 (9.6)	5 (8.6)
The left lung	Upper lobe	9 (50)	25 (71.4)	24 (53.3)
	Lower lobe	10 (55.6)	10 (28.6)	18 (40)
	Lung hilum	1 (5.6)	2 (5.7)	5 (11.1)

Values are given as *n* (%).

**Table 3:** Grading of the primary lung cancer in each group.

Grade	Group 1a	Group 1b	Group 2
G1	13 (25.0)	24 (27.6)	23 (22.3)
G2	23 (44.2)	24 (27.6)	25 (24.3)
G3	4 (7.7)	15 (17.2)	20 (19.4)
0	12 (23.1)	24 (27.6)	35 (34.0)
Total	52 (100.0)	87 (100.0)	103 (100.0)

Values are given as *n* (%).

**Table 4:** TNM VII stage.

Stage TNM VII	Group 1a	Group 1b	Group 2
	*n* = 52	*n* = 87	*n* = 103
T1	28 (53.8)	24 (27.6)	35 (34.0)
T2	14 (26.9)	34 (39.1)	41 (39.8)
T3	6 (11.5)	19 (21.8)	17 (16.5)
T4	4 (7.7)	10 (11.5)	10 (9.7)
N0	42 (80.8)	62 (71.3)	82 (79.6)
N1	2 (3.8)	6 (6.9)	2 (1.9)
N2	6 (11.5)	16 (18.4)	14 (13.6)
N3	1 (1.9)	0 (0.0)	1 (1.0)
Nx	1 (1.9)	3 (3.4)	4 (3.9)
M1b	1 (1.9)	1 (1.1)	0 (0.0)
Mx	1 (1.9)	6 (6.9)	4 (3.9)

Values are given as *n* (%).

**Table 5:** Comparison of the frequency of features from T1a to T2b according to the TNM VII classification in groups 1a and 1b.

TNM VII Stage	Group 1a	Group 1b	*p*-Value
T1a	21 (58.3)	13 (26.5)	0.002
T1b	7 (19.4)	11 (22.4)	0.9
T2a	4 (11.1)	19 (38.8)	0.053
T2b	4 (11.1)	6 (12.2)	0.19

Values are given as *n* (%).

**Table 6:** Comparison of the frequency of features from T1a to T2b according to the TNM VII classification in groups 1a and 2.

TNM VII Stage	Group 1a	Group 2	*p*-Value
T1a	21 (58.3)	16 (25.4)	0.001
T1b	7 (19.3)	19 (30.2)	0.58
T2a	4 (11.1)	20 (31.7)	0.09
T2b	4 (11.1)	8 (12.7)	0.23

Values are given as *n* (%).

**Table 7:** Comparison of the frequency of features from T1a to T2b according to the TNM VII classification in groups 1b and 2.

TNM VII Stage	Group 1b	Group 2	*p*-Value
T1a	13 (26.5)	16 (25.4)	0.93
T1b	11 (22.4)	19 (30.2)	0.37
T2a	19 (38.8)	20 (31.7)	0.82
T2b	6 (12.2)	8 (12.7)	0.96

Values are given as *n* (%).

**Table 8:** Clinical stages of the primary lung cancer in the three study groups based on the TNM VII classification.

Clinical Stage	Group 1a *n* = 52	Group 1b *n* = 87	Group 2 *n* = 103
IA	26 (50)	21 (24.1)	33 (32.0)
IB	6 (11.5)	19 (21.8)	25 (24.3)
IIA	5 (9.6)	8 (9.2)	9 (8.7)
IIB	3 (5.8)	10 (11.5)	16 (15.5)
IIIA	9 (17.3)	26 (29.9)	15 (14.6)
IIIB	2 (3.8)	2 (2.3)	5 (4.9)
IV	1 (1.9)	1 (1.1)	0 (0.0)

Values are given as *n* (%).

**Table 9:** Characteristics of tumor volume in patients in groups 1a, 1b, and 2.

Tumor Volume (cm^3^)	Group 1a	Group 1b	Group 2	Combined Groups 1b i 2
min–max	0.112–309	0.027–403.2	0.1–800.7	0.027–800.7
Q_1_–Q_3_	1.89–35.8	7.92–64.0	6.91–70.2	7.04–64.0
m_e_ (Q)	9.648 (16.97)	23.4 (28.04)	20.46 (31.644)	21.48 (28.48)
GM geometric mean	8.09	21.22	19.97	20.53
$\bar{x}$ (SD)	35.96 (64.58)	59.6 (83.0)	75.1 (141.3)	68.0 (118.2)

Values are given as *n* (%).

**Table 10:** Comparison of the number of diagnoses by invasive procedures in the studied groups.

Group	Diagnosis via Invasive Procedure		*p*-Value
	No. Achieved (%)	No. Not Achieved (%)	
1a	20 (38.5%)	32 (61.5%)	>0.1
1b	43 (49.4%)	44 (50.6%)	>0.8
2	56 (54.4%)	47 (45.6%)	>0.2

**Table 11:** Statistical comparison of the procedures performed in the studied groups.

Type of Procedure	Significance Level in the Compared Groups			
	1a vs. 1b	1a vs. 2	1b vs. 2	1a vs. (1b + 2)
Lobectomy	*p* = 0.02	*p* = 0.04	*p* = 0.74	*p* = 0.01
Wedge resection	*p* = 0.08	*p* = 0.65	*p* = 0.14	*p* = 0.22
Segmentectomy	*p* = 0.83	*p* = 0.33	*p* = 0.29	*p* = 0.55
Bilobectomy	*p* = 0.83	*p* = 0.89	*p* = 0.96	*p* = 0.95
Pneumonectomy	*p* = 0.32	*p* = 0.33	*p* = 0.9	*p* = 0.27

## Data Availability

Data available on request due to ethical reasons. Patient’s records are kept in the archives of the Department of Thoracic Surgery and Transplantation, Pomeranian Medical University, Szczecin, Poland.

## References

[1] Siegel R.L., Kratzer T.B., Giaquinto A.N., Sung H., Jemal A. (2025). Cancer statistics, 2025. CA Cancer J. Clin..

[2] Danila E., Krynke L., Ciesiūnienė A., Žučenkienė E., Kantautas M., Gricienė B., Valančienė D., Zeleckienė I., Austrotienė R., Tarutytė G. (2025). The Lithuanian Lung Cancer Screening Model: Results of a Pilot Study. Cancers.

[3] Souliotis K., Golna C., Golnas P., Markakis I.-A., Linardou H., Sifaki-Pistolla D., Hatziandreou E. (2022). Lung Cancer Screening in Greece: A Modelling Study to Estimate the Impact on Lung Cancer Life Years. Cancers.

[4] Krzakowski M., Jassem J., Antczak A., Błasińska K., Chorostowska-Wynimko J., Dziadziuszko R., Głogowski M., Grodzki T., Kowalski D., Krenke R. (2022). Nowotwory klatki piersiowej. Onkol. Prakt. Klin.—Eduk..

[5] Didkowska J., Wojciechowska U., Zatoński W. (2012). Nowotwory Złośliwe w Polsce w 2010 Roku.

[6] Raz D.J., Wu G.X., Consunji M., Nelson R., Sun C., Erhunmwunsee L., Ferrell B., Sun V., Kim J.Y. (2016). Perceptions and Utilization of Lung Cancer Screening Among Primary Care Physicians. J. Thorac. Oncol..

[7] Rzyman W. (2008). Rak płuca. Forum Med. Rodz..

[8] Shimamoto T., Tateyama Y., Kobayashi D., Yamamoto K., Nishioka N., Takahashi Y., Ueshima H., Sasaki K., Kiyohara K., Nakayama T. (2025). Observational Study on Actual Cancer Screening Participation and Outcomes Among Patients with Lung Cancer Based on Linkage of Cancer Registry and Kyoto City Integrated Database Data from 2014 to 2018. Int. J. Environ. Res. Public Health.

[9] Grodzki T., Walecka A., Fabian W., Daniel B., Witkiewicz I., Jarmoliński T., Alchimowicz J., Wójcik J. (2009). Program wczesnego wykrywania nowotworów płuc za pomocą tomografii komputerowej—Wstępne doświadczenia Szczecina. Pneumonol. Alergol. Pol..

[10] Kowalski D.M., Knetki-Wróblewska M., Krawczyk P., Kuncman Ł., Langfort R., Antczak A., Bryl M., Dziadziuszko R., Fijuth J., Jaśkiewicz P. (2025). Recommendations of the Expert Panel and the Polish Lung Cancer Study Group on the use of chemotherapy in combination with osimertinib for treatment of non-small cell lung cancer patients with pathogenic variants of the EGFR gene. Oncol. Clin. Pract..

[11] Wang W., Song Z., Zhang Y. (2018). Efficacy of brain radiotherapy plus EGFR-TKI for EGFR-mutated non-small cell lung cancer patients who develop brain metastasis. Arch. Med. Sci..

[12] Gasparri R., Sabalic A., Spaggiari L. (2023). The Early Diagnosis of Lung Cancer: Critical Gaps in the Discovery of Biomarkers. J. Clin. Med..

[13] Parker M.S., Groves R.C., Shepherd R.W., Cassano A.D., Cafaro P.L., Chestnut G.T. (2014). Lung cancer screening with low-dose computed tomography: An analysis of the MEDCAC decision. J. Thorac. Imaging.

[14] Lang-Lazdunski L. (2013). Surgery for nonsmall cell lung cancer. Eur. Respir. Rev..

[15] Ruano-Ravina A., Heleno B., Fernandez-Villar A. (2014). Lung cancer screening with low-dose CT (LDCT), or when a public health intervention is beyond the patient’s benefit. J. Epidemiol. Community Health.

[16] National Lung Screening Trial Research Team (2011). The National Lung Screening Trial: Overview and study design. Radiology.

[17] Vansteenkiste J., Dooms C., Mascaux C., Nackaerts K. (2012). Screening and early detection of lung cancer. Ann. Oncol..

[18] Orłowski T. (2014). [Early lung cancer—The role of screening programs]. Pneumonol. Alergol. Pol..

[19] Zhao Y.R., Xie X., de Koning H.J., Mali W.P., Vliegenthart R., Oudkerk M. (2011). NELSON lung cancer screening study. Cancer Imaging.

[20] Yousaf-Khan U., van der Aalst C., de Jong P.A., Heuvelmans M., Scholten E., Walter J., Nackaerts K., Groen H., Vliegenthart R., Haaf K.T. (2017). Risk stratification based on screening history: The NELSON lung cancer screening study. Thorax.

[21] Veronesi G., Baldwin D.R., Henschke C.I., Ghislandi S., Iavicoli S., Oudkerk M., De Koning H.J., Shemesh J., Field J.K., Zulueta J.J. (2020). Recommendations for Implementing Lung Cancer Screening with Low-Dose Computed Tomography in Europe. Cancers.

[22] Benzaquen J., Boutros J., Marquette C., Delingette H., Hofman P. (2019). Lung Cancer Screening, towards a Multidimensional Approach: Why and How?. Cancers.

[23] Lee W.-Y., Chen P.-H., Chen K.-C., Hsu H.-H., Chen J.-S. (2022). Computed Tomography-Guided Localization and Extended Segmentectomy for Non-Small Cell Lung Cancer. Diagnostics.

[24] Malbasa J.D., Kovacevic T., Vukoja M., Bursać D., Bokan D., Stojšić V., Zaric B. (2025). Nodule Characteristics, Clinical Risk Factors, and Radiologist Experience as Predictors of Positive Baseline LDCT Screening Results. Healthcare.

[25] Hunger T., Wanka-Pail E., Brix G., Griebel J. (2021). Lung Cancer Screening with Low-Dose CT in Smokers: A Systematic Review and Meta-Analysis. Diagnostics.

[26] Aberle D.R. (2017). Implementing lung cancer screening: The US experience. Clin. Radiol..

[27] Fu S.S., Rothman A.J., Vock D.M., Lindgren B., Almirall D., Begnaud A., Melzer A., Schertz K., Glaeser S., Hammett P. (2017). Program for lung cancer screening and tobacco cessation: Study protocol of a sequential, multiple assignment, randomized trial. Contemp. Clin. Trials.

[28] Zhu J., Branstetter S., Lazarus P., Muscat J.E. (2024). Smoking, Lung Cancer Stage, and Prognostic Factors—Findings from the National Lung Screening Trial. Int. J. Environ. Res. Public Health.

[29] Kang H.-R., Song J.H., Kim Y.W., Chung K.B., Cho S., Jang S.H., Chung J.-H., Lee J., Lee C.-T. (2025). Single-Round LDCT Screening in Men Aged ≥ 70 Years: Prevalence of Pulmonary Nodules and Lung Cancer Detection. Cancers.

[30] Warner E., Jotkowitz A., Maimon N. (2010). Lung cancer screening—Are we there yet?. Eur. J. Intern. Med..

[31] Gonzalez M., Vignaud J.-M., Clement-Duchene C., Luc A., Wild P., Bertrand O., Thiberville L., Martinet Y., Benichou J., Paris C. (2012). Smoking, occupational risk factors, and bronchial tumor location. A possible impact for lung cancer computed tomography scan screening. J. Thorac. Oncol..

[32] Becker N., Motsch E., Gross M.L., Eigentopf A., Heussel C., Dienemann H., Schnabel P., Eichinger M., Optazaite D.-E., Puderbach M. (2015). Randomized Study on Early Detection of Lung Cancer with MSCT in Germany: Results of the First 3 Years of Follow-up After Randomization. J. Thorac. Oncol..

[33] Aberle D.R., Adams A.M., Berg C.D., Black W.C., Clapp J.D., Fagerstrom R.M., Gareen I.F., Gatsonis C., Marcus P.M., Fagerstrom R.M. (2013). Results of the two incidence screenings in the National Lung Screening Trial. N. Engl. J. Med..

[34] Kondo R., Yoshida K., Kawakami S., Shiina T., Kurai M., Takasuna K., Yamamoto H., Koizumi T., Honda T., Kubo K. (2011). Different efficacy of CT screening for lung cancer according to histological type: Analysis of Japanese-smoker cases detected using a low-dose CT screen. Lung Cancer.

[35] Voigt W., Prosch H., Silva M. (2023). Clinical Scores, Biomarkers and IT Tools in Lung Cancer Screening—Can an Integrated Approach Overcome Current Challenges?. Cancers.

[36] Sawabata N., Asamura H., Goya T., Mori M., Nakanishi Y., Eguchi K., Koshiishi Y., Okumura M., Miyaoka E., Fujii Y. (2010). Japanese Lung Cancer Registry Study: First prospective enrollment of a large number of surgical and nonsurgical cases in 2002. J. Thorac. Oncol..

[37] Heuvelmans M.A., Vliegenthart R., Oudkerk M. (2015). Contributions of the European trials (European randomized screening group) in computed tomography lung cancer screening. J. Thorac. Imaging.

[38] Eggeling S., Martin T., Böttger J., Beinert T., Gellert K. (2002). Invasive staging of non-small cell lung cancer—A prospective study. Eur. J. Cardiothorac. Surg..

[39] Cressman S., Lam S., Tammemagi M.C., Evans W.K., Leighl N.B., Regier D.A., Bolbocean C., Shepherd F.A., Tsao M.-S., Manos D. (2014). Resource utilization and costs during the initial years of lung cancer screening with computed tomography in Canada. J. Thorac. Oncol..

[40] Sharples L.D., Jackson C., Wheaton E., Griffith G., Annema J., Dooms C., Tournoy K., Deschepper E., Hughes V., Magee L. (2012). Clinical effectiveness and cost-effectiveness of endobronchial and endoscopic ultrasound relative to surgical staging in potentially resectable lung cancer: Results from the ASTER randomised controlled trial. Health Technol. Assess..

[41] Tammemägi M.C., Katki H.A., Hocking W.G., Church T.R., Caporaso N., Kvale P.A., Chaturvedi A.K., Silvestri G.A., Riley T.L., Commins J. (2013). Selection criteria for lung-cancer screening. N. Engl. J. Med..

[42] Walker B.L., Williamson C., Regis S.M., McKee A.B., D’agostino R.S., Hesketh P.J., Lamb C.R., Flacke S., Wald C., McKee B.J. (2015). Surgical Outcomes in a Large, Clinical, Low-Dose Computed Tomographic Lung Cancer Screening Program. Ann. Thorac. Surg..

[43] Wiener R.S., Schwartz L.M., Woloshin S., Welch H.G. (2011). Population-based risk for complications after transthoracic needle lung biopsy of a pulmonary nodule: An analysis of discharge records. Ann. Intern. Med..

[44] Knorst M.M., Dienstmann R., Pankowski-Fagundes L. (2003). Delay in the diagnosis and surgical treatment of lung cancer. J. Pneumol..

[45] Jensen A.R., Mainz J., Overgaard J. (2002). Impact of delay on diagnosis and treatment of primary lung cancer. Acta Oncol..

[46] Pitz C.C., de la Rivière A.B., van Swieten H.A., Westermann C.J., Lammers J.W., van den Bosch J.M. (2003). Results of surgical treatment of T4 non-small cell lung cancer. Eur. J. Cardiothorac. Surg..

[47] Petersen R.H., Hansen H.J., Dirksen A., Pedersen J.H. (2012). Lung cancer screening and video-assisted thoracic surgery. J. Thorac. Oncol..

[48] Alarcón J.P., Cuesta J.C.P. (2013). Experience with lung resection in a fast-track surgery program. Arch. Bronconeumol..

[49] Hennon M.W., Yendamuri S. (2012). Advances in lung cancer surgery. J. Carcinog..

[50] Dominioni L., Rotolo N., Poli A., Castiglioni M., Mangini M., Spagnoletti M., Paolucci M., Paddeu A., Mantovani W., Zanini A. (2013). Cost of a population-based programme of chest x-ray screening for lung cancer. Monaldi Arch. Chest Dis..

[51] Shiono S., Abiko M., Sato T. (2013). Postoperative complications in elderly patients after lung cancer surgery. Interact. Cardiovasc. Thorac. Surg..

[52] Little A.G., Rusch V.W., Bonner J.A., Gaspar L.E., Green M.R., Webb W.R., Stewart A.K. (2005). Patterns of Surgical Care of Lung Cancer Patients. Ann. Thorac. Surg..

[53] Thomas P., Michelet P., Barlesi F., Thirion X., Doddoli C., Giudicelli R., Fuentes P. (2007). Impact of blood transfusions on outcome after pneumonectomy for thoracic malignancies. Eur. Respir. J..

[54] Parikh S., Bentz T., Crowley S., Greenspan S., Costa A., Bergese S. (2025). Perioperative Blood Management. J. Clin. Med..

[55] Gagarine A., Urschel J.D., Miller J.D., Bennett W.F., Young J.E. (2003). Preoperative and intraoperative factors predictive of length of hospital stay after pulmonary lobectomy. Ann. Thorac. Cardiovasc. Surg..

[56] Ueda K., Murakami J., Tanaka T., Hayashi M., Okabe K., Hamano K. (2018). Preoperative risk assessment with computed tomography in patients undergoing lung cancer surgery. J. Thorac. Dis..

[57] Cressman S., Peacock S.J., Tammemägi M.C., Evans W.K., Leighl N.B., Goffin J.R., Tremblay A., Liu G., Manos D., MacEachern P. (2017). The Cost-Effectiveness of High-Risk Cancer Screening and Drivers of Program Efficiency. J. Thoracic. Oncol..

[58] Carozzi F.M., Bisanzi S., Carrozzi L., Falaschi F., Pegna A.L., Mascalchi M., Picozzi G., Peluso M., Sani C., Greco L. (2017). Multimodal lung cancer screening using the ITALUNG biomarker panel and low dose computed tomography. Results of the ITALUNG biomarker study. Int. J. Cancer.

[59] Cotton L.B., Bach P.B., Cisar C., Schonewolf C.A., Tennefoss D., Vachani A., Carter-Bawa L., Zaidi A.H. (2024). Innovations in Early Lung Cancer Detection: Tracing the Evolution and Advancements in Screening. J. Clin. Med..

[60] Zheng S., Kong S., Huang Z., Pan L., Zeng T., Zheng B., Yang M., Liu Z. (2022). A Lower False Positive Pulmonary Nodule Detection Approach for Early Lung Cancer Screening. Diagnostics.

[61] Zarinshenas R., Amini A., Mambetsariev I., Abuali T., Fricke J., Ladbury C., Salgia R. (2023). Assessment of Barriers and Challenges to Screening, Diagnosis, and Biomarker Testing in Early-Stage Lung Cancer. Cancers.

[62] Jiang J.-M., Miao L., Liang X., Liu Z.-H., Zhang L., Li M. (2022). The Value of Deep Learning Image Reconstruction in Improving the Quality of Low-Dose Chest CT Images. Diagnostics.

[63] Chao H.-S., Tsai C.-Y., Chou C.-W., Shiao T.-H., Huang H.-C., Chen K.-C., Tsai H.-H., Lin C.-Y., Chen Y.-M. (2023). Artificial Intelligence Assisted Computational Tomographic Detection of Lung Nodules for Prognostic Cancer Examination: A Large-Scale Clinical Trial. Biomedicines.

